# Effectiveness of Protease Inhibitor Monotherapy versus Combination Antiretroviral Maintenance Therapy: A Meta-Analysis

**DOI:** 10.1371/journal.pone.0022003

**Published:** 2011-07-19

**Authors:** Sandra Mathis, Bettina Khanlari, Federico Pulido, Mauro Schechter, Eugenia Negredo, Mark Nelson, Pietro Vernazza, Pedro Cahn, Jean-Luc Meynard, Jose Arribas, Heiner C. Bucher

**Affiliations:** 1 Basel Institute for Clinical Epidemiology and Biostatistics, University Hospital Basel, Basel, Switzerland; 2 Division of Infectious Diseases and Hospital Hygiene, University Hospital Basel, Basel, Switzerland; 3 HIV Unit, Hospital 12 de Octubre, i+12, Universidad Complutense de Madrid, Madrid, Spain; 4 Departement de Medicina Preventiva da Facultade de Medicina, Universida Federal do Rio de Janeiro, Rio de Janeiro, Brasil; 5 Fundació Lluita Contra la Sida, Hospital Universitari Germans Trias i Pujol, Badalona, Barcelona, Spain; 6 Chelsea and Westminster Hospital, London, United Kingdom; 7 Division of Infectious Diseases, Kantonsspital St. Gallen, St. Gallen, Switzerland; 8 Fundacion Huesped, Buenos Aires, Argentina; 9 Service de Maladies Infectieuses et Tropicales, Hôpital Saint-Antoine, Paris, France; 10 Servicio de Medicina Interna, Hospital la Paz, Universidad Autonoma de Madrid, IdiPAZ, Madrid, Spain; University of Toronto, Canada

## Abstract

**Background:**

The unparalleled success of combination antiretroviral therapy (cART) is based on the combination of three drugs from two classes. There is insufficient evidence whether simplification to ritonavir boosted protease inhibitor (PI/r) monotherapy in virologically suppressed HIV-infected patients is effective and safe to reduce cART side effects and costs.

**Methods:**

We systematically searched Medline, Embase, the Cochrane Library, conference proceedings and trial registries to identify all randomised controlled trials comparing PI/r monotherapy to cART in suppressed patients. We calculated in an intention to treat (loss-of follow-up, discontinuation of assigned drugs equals failure) and per-protocol analysis (exclusion of protocol violators following randomisation) and based on three different definitions for virological failure pooled risk ratios for remaining virologically suppressed.

**Findings:**

We identified 10 trials comparing 3 different PIs with cART based on a PI/r plus 2 reverse transcriptase inhibitors in 1189 patients. With the most conservative approach (viral load <50 copies/ml on two consecutive measurements), the risk ratios for viral suppression at 48 weeks of PI/r monotherapy compared to cART were in the ITT analysis 0.94 8 (95% CI 0.89 to 1.00) p = 0.06; risk difference −0.06 (95%CI -0.11 to 0) p = 0.05, p for heterogeneity  = 0.08, I^2^ = 43.1%) and in the PP analysis 0.93 ((95%CI 0.90 to 0.97) p<0.001; risk difference −0.07 (95%CI −0.10 to −0.03) p<0.001, p for heterogeneity  = 0.44, I^2^ = 0%). Reintroduction of cART in 44 patients with virological failure led in 93% to de-novo viral suppression.

**Interpretation:**

Virologically well suppressed HIV-infected patients have a lower chance to maintain viral suppression when switching from cART to PI/r monotherapy. Failing patients achieve high rates of de-novo viral suppression following reintroduction of reverse transcriptase inhibitors.

## Introduction

Modern combination antiretroviral therapy (cART) for HIV-infected drug naïve patients consists of a combination of three antiretroviral drugs from two classes, typically a boosted protease inhibitor or non-nucleoside analogue (NNTRI) in combination with two reverse transcriptase inhibitors (NRTI) [1]. Despite the remarkable success of dual class based cART [2] and the availability of multiple compound formulations allowing once daily intake with low pill burden[3], the concept of treating HIV-infected individuals with one single very potent drug for simplified maintenance therapy has attracted clinical HIV researchers over the past years [4], [5]. The rational for this therapeutic approaches is the potential advantage of reduced adverse drug reactions, drug-drug interactions, reduced costs and the preservation of future treatment options in case of resistance related drug failure.

Ritonavir boosted protease inhibitors (PI/r) like, lopinavir, atazanavir, saquinavir and darunavir are candidates for maintenance mono-therapy due to their high potency and genetic barrier for drug resistance and possibility for once daily dosing. Several controlled and uncontrolled studies have been conducted to examine the safety and tolerance of PI/r monotherapy for maintenance in HIV-infected patients. Many of these studies were small or did not use controls and evidence on the efficacy and safety of PI/r monotherapy is therefore limited [6]. Treatment of HIV infected patients with PI/r monotherapy for maintenance is experimental [7] and guidelines consider PI/r monotherapy only for selective patient groups [8]. However, more evidence of the effectiveness, the potential risk of drug failure and PI resistance is needed to better define the future role of PI/r mono-maintenance therapy.

We present a systematic review and meta-analysis of randomized controlled trials to examine the effectiveness and safety of antiretroviral maintenance therapy of PI/r monotherapy in comparison with continued dual class PI/r and NRTI based cART in virologically suppressed HIV-infected patients.

## Methods

### Literature search

We searched Medline, Embase, Pascal, Biological abstracts, Web of Science and the Cochrane Central Register of Controlled Trials from January 1996 through August 2010 for all randomized controlled trials comparing PI-monotherapy to conventional cART with the aid of a librarian. The following search terms were used: (“Protease inhibitors (Mesh) OR “antiretroviral substance” or monotherapy (textword) OR saquinavir OR indinavir OR lopinavir OR ritonavir OR amprenavir OR atazanavir OR darunavir] AND (random (text word) or randomized controlled trials (publication type)) and (limited to humans). Two reviewers (SM and BK) independently searched reference lists of identified articles, recently published editorials and reviews on the topic for further eligible trials. They additionally checked abstracts of all relevant conferences (Conference on HIV Pathogenesis, Treatment and Prevention (IAS), International AIDS Conference, European AIDS Conference (EACS), Conference on Retroviruses and Opportunistic Infections (CROI), International Congress on Drug Therapy in HIV Infection, Glasgow) and the three trial registries of planned or ongoing clinical trials by the US Institutes of Health (http://clinicaltrials.gov), Current Controlled Trials (http://www.controlled-trials.com), and the WHO International Clinical Trials Registry Platform (http://apps.who.int/trialsearch) from their inception though August 2010 for trials that involved any of the PIs mentioned above. We used no language restriction. Authors of included primary trials were contacted for the identification of additional unpublished trials and for the contribution of additional data relevant for the purpose of this analysis.

### Trial Selection

Two reviewers (SM and BK) independently checked all studies for eligibility, disagreement was resolved by consensus. Trials were eligible if they fulfilled the following criteria: Randomized controlled trials comparing ritonavir boosted PI monotherapy with cART consisting either of a PI/r or NNRTI combined with two NRTIs, patients at randomisation had to be virologically suppressed for at least 6 months (based on the trial specific definition for viral suppression) and trials had to report virological outcome data at ≥24 weeks. We excluded all trials that used unboosted PIs, sequentially introduced NRTIs, or randomized cART naïve patients to PI/r monotherapy.

### Validity assessment

The quality of each included trial was independently assessed by the two reviewers for concealment of treatment allocation, blinding of patients, caregivers, or clinical outcome assessors, and for the proportion of patients with complete clinical follow-up. Treatment assignment was considered concealed if allocation was centrally done by an independent randomisation facility and we considered a loss of follow-up of >10% as insufficient.

### Data abstraction and outcome variables

All data extraction was done independently by two reviewers. When information from the studies was missing, incomplete or in a format that did not allow for pooling, the authors from the original trials were contacted for additional data. Data abstractors were not blinded in regard to the source and authorship of published trial data. Endpoints and adverse events were considered irrespective of their putative relation to the treatment.

The primary endpoint for this analysis was the proportion of patients with maintained virological suppression with failure as defined in individual trials, HI viral loads >50 copies/ml and >500 copies/ml in plasma on two consecutive measurements two weeks apart at ≥24 weeks of follow-up. Secondary endpoints were AIDS or death and the proportion of patients with genotypic resistance mutations.

### Quantitative data synthesis

We pooled treatment effects and calculated risk ratios and risk differences for remaining virologically suppressed with three different definitions for virological failure and used a random effects model [9]. We explored heterogeneity with the Cochran Q test and measured inconsistency (I^2^ the percentage of total variance across studies that is due to heterogeneity rather than chance) of treatment effects across all endpoints [10], [11]. We investigated the presence of publication bias with funnel plots [12]. In the intention to treat analysis patients with missing data, loss to follow-up or discontinuation of assigned treatment for any reason were considered as failure. In the per-protocol analysis we analyzed the proportions of individuals with virological failure from all individuals randomized to either PI/r or cART by excluding patients with discontinuation of the assigned treatment, loss-of follow-up or drop outs. Patients in PI/r with virological failure and successful reintroduction of NRTIs were disregarded in our per-protocol analysis and considered as permanent failures. We did not a priori define a non-inferiority margin for this analysis because this approach remains arbitrary and based on inference entirely borrowed from external data [13], [14]. We conducted a sensitivity analysis to examine treatment effects according to quality components of included trials (concealed treatment allocation and sample size calculation for non-inferiority) and the type of PI. We used Stata 10 (StataCorp, College Station/Texas) for data analysis.

## Results

### Trial selection process

**Figure 1:**
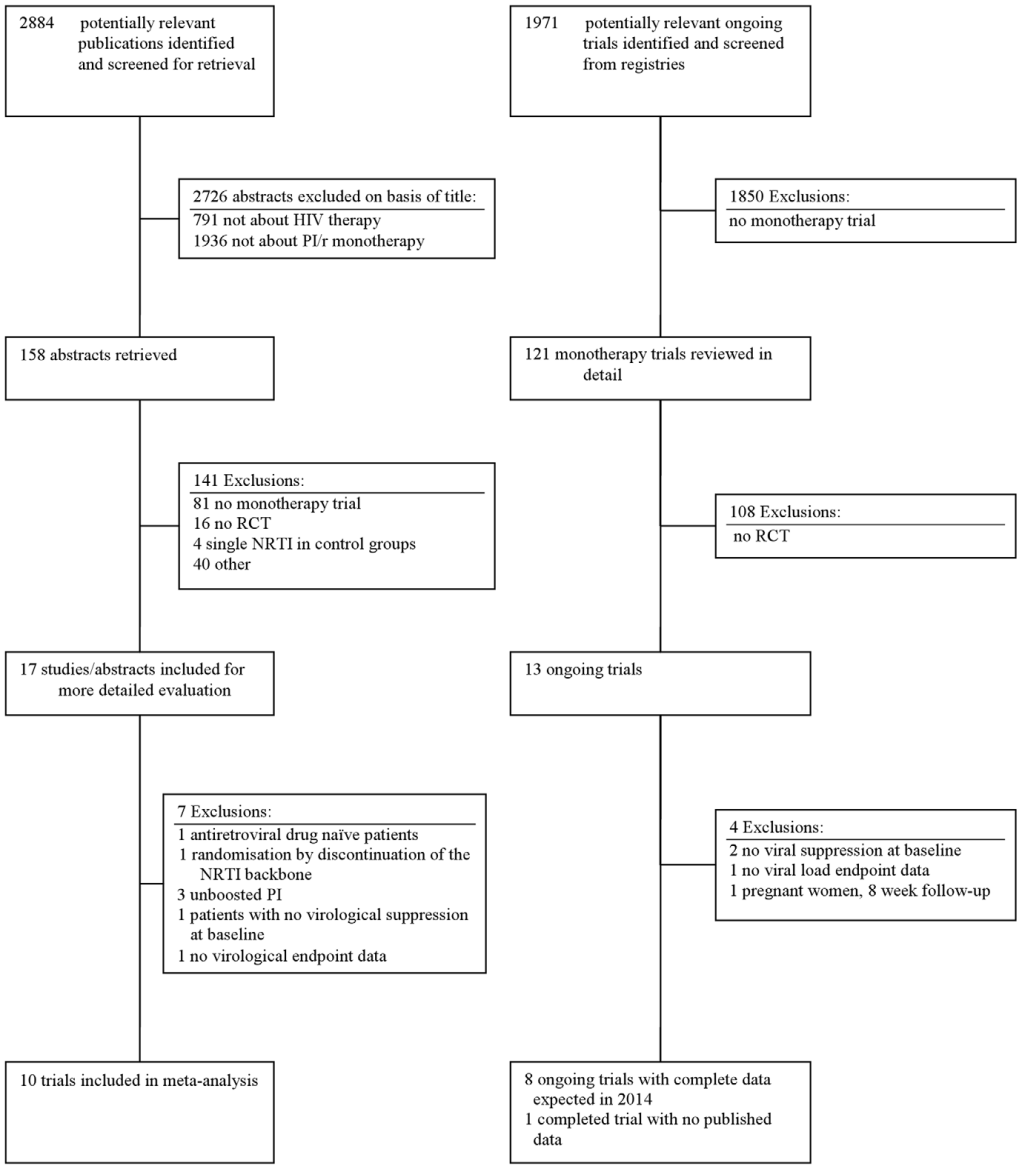
Flow chart for the identification of eligible trials for boosted proteinase inhibitor mono therapy in HIV infection.

We screened 2884 references, 2726 references could be excluded on the basis of the title. The remaining 158 studies were reviewed in detail (Figure 1). Of these studies, 141 references were either not randomized controlled trials or included no PI/r monotherapy arm. We excluded all trials that compared full dose ritonavir and saquinavir (each 400 mg bid) with a single NRTI backbone [15]–[17]. We identified 17 randomized controlled trials and excluded 7 trials for the following reasons: One trial included naïve patients [18], one trial initiated PI/r monotherapy following randomisation by discontinuation of the NRTI backbone [19], three trials were conducted with unboosted PIs using mono or dual NRTI as the comparator regimen [20]–[22], one trial was conducted in viremic patients [23] and one trial reported no virological endpoint data [24]. We identified 13 ongoing trials and of these four do not qualify for the following reasons: Two trials recruit patients not virologically suppressed at study entry [25], [26], one trial does not provide virological endpoint data [27], and one trial investigates a prepartum simplification strategy for 8 weeks in pregnant women to prevent mother to child HIV transmission [28]. Eight ongoing trials formally fulfil our inclusion criteria [29]–[36]. Six trials use boosted lopinavir, one trial uses a mixture of PIs [35], and one trial uses darunavir [36]. These trials will terminate data collection with 48 week follow-up data by end of 2012 for about 450 of 1290 anticipated study participants. Of these, three trials recruit about 160 co-infected patients with concomitant antiviral treatment of hepatitis C [33], [34], [37] and will provide data by mid 2011. One trial was completed in 2008 but never published [38]. Ten trials fulfilled all criteria and were included into this analysis.

### Baseline characteristics of included trials

The ten trials included a total of 1189 patients, 590 patients treated with PI monotherapy and 599 patients with continued cART and a total of 1094 patients for a per-protocol analysis (Table S1). Seven trials used ritonavir boosted lopinavir [4], [39]–[44], two boosted darunavir[45], [46] and one boosted saquinavir [47]. In all trials the PI/r in the cART regimen was the same as in the monotherapy arm. One trial investigated virological failure in cerebral spinal fluid and serum and was stopped prematurely due to increased virological failures in PI/r monotherapy recipients [41]. For this analysis we used only failure data from the plasma. The trials were generally small and the number of enrolled individuals varied between 28 to 256 patients[46], [47]. Six trials had a follow up of 48 to 52 weeks [39], [41], [43]–[45], [47], one trial had 72 [4], [48] and three trials [40], [42], [46], [49], [50] had 96 weeks of follow-up.

In all trials patients had to be on cART for at least 6 months with suppressed plasma viral load (i.e. <50 copies/ml, <80 copies/ml in one trial [42]) at randomisation. The mean age of enrolled subjects in individual studies was about 40 years and the percentage of enrolled males and IV drug users was between 55% and 100% and between 29% and 46%, respectively. Four trials described concealed allocation of patients, in the remaining trials this information was missing. The extent of follow-up in individual studies was good, and all trials had less than 10% patients lost to follow-up. All trials were open interventions with no blinded endpoint assessment. Adequate information for power calculations was available from five trials. The relatively small number of trials precluded a sensitive exploration of publication bias, although the plots of standardized effect against precision for primary outcomes did not indicate evidence for such a bias (Figures S1, S2, S3, S4, S5, S6).

### Loss of virological suppression

The studies used different definitions for virological failure. Two trials used a cut-off <500 copies/ml[4], [40], two trials <400 copies/ml [41], [45], one trial <200 copies/ml [43], one trial<80 copies/ml [42], and four trials <50 copies/ml [39], [44], [46], [47] (Table S2). Nine trials provided virological failure data with a cut-off <50 copies/ml.

**Figure 2:**
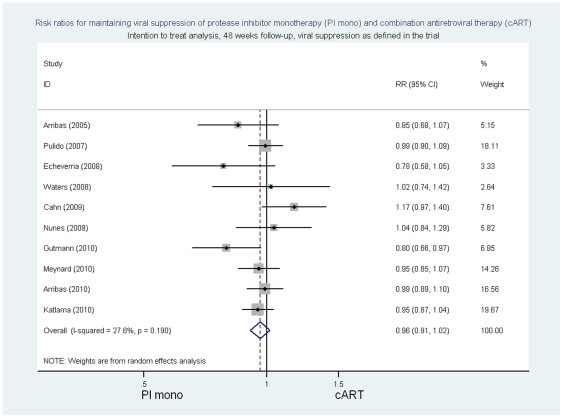
Risk ratios for maintaining viral suppression, intention to treat analysis, 48 week follow-up, viral suppression as defined in the trial.

**Figure 3:**
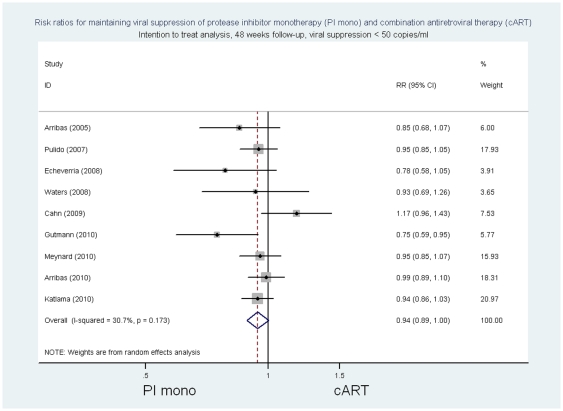
Risk ratios for maintaining viral suppression, intention to treat analysis, 48 week follow-up, viral suppression <50 copies/ml.

In the intention to treat analysis, the summary risk ratio at 48 weeks of follow-up of PI/r monotherapy compared to cART for viral suppression as defined in individual trials was 0.96 ((95%CI 0.91 to 1.02) p = 0.18, p for heterogeneity 0.19, I^2^ = 27.6%; risk difference −0.04 (95%CI −0.09 to 0.02) p = 0.16, p for heterogeneity  = 0.08, I^2^ = 41.2%) (Figure 2 and Table S3). The respective risk ratios of PI/ monotherapy compared with cART for viral suppression with <50 copies/ml were 0.94 [(95% CI 0.89 to 1.00) p = 0.06 p for heterogeneity 0.17 I^2^ = 30.7%; risk difference −0.06 (95%CI -0.11 to 0) p = 0.05, p for heterogeneity  = 0.08, I^2^ = 43.1%] (Figure 3 and Table S3) and for viral suppression with <500 copies/ml 0.98 [(95%CI 0.93 to 1.03) p>0.20, p for heterogeneity 0.18, I^2^ = 29.9%; risk difference −0.02 (95%CI -0.08 to 0.03) p>0.20, p for heterogeneity  = 0.10, I^2^ = 39.6].

**Figure 4:**
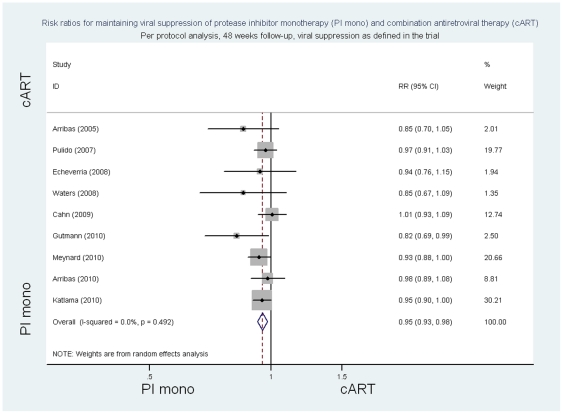
Risk ratios for maintaining viral suppression, per protocol analysis, 48 week follow-up, viral suppression as defined in the trial.

**Figure 5:**
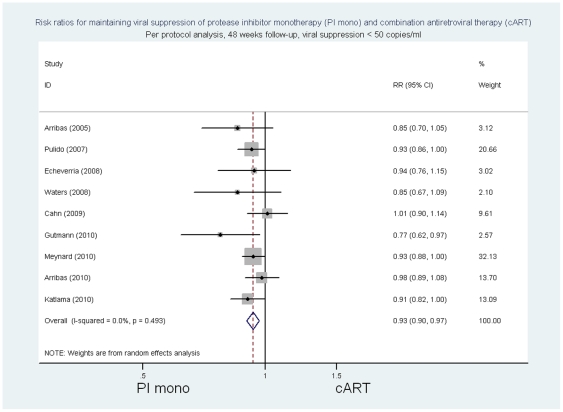
Risk ratios for maintaining viral suppression, per protocol analysis, 48 week follow-up, viral suppression <50 copies/ml.

In the per protocol analysis, the summary risk ratios at 48 weeks of follow-up of PI/r monotherapy compared to cART for viral suppression as defined in individual trials was 0.95 ((95%CI 0.93 to 0.98) p = 0.001, p for heterogeneity 0.49 I^2^ = 0%; risk difference −0.05 (95%CI −0.08 to −0.02) p = 0.001, p for heterogeneity  = 0.42, I^2^ = 2.2%) (Figure 4 and Table S3). The risk ratios of PI/monotherapy compared with cART for viral suppression with <50 copies/ml were 0.93 ((95% CI 0.90 to 0.97) p<0.001, p for heterogeneity 0.49 I^2^  = 0%; risk difference -0.07 (95%CI −0.10 to 0.03) p<0.001, p for heterogeneity  = 0.44, I^2^ = 0%) (Figure 5 and Table S3) and for viral suppression <500 copies/ml 0.97 ((95%CI 0.93 to 1.0) p = 0.06, p for heterogeneity 0.12 I^2^ = 39.4%; risk difference −0.04 (95%CI −0.07 to 0) p = 0.04, p for heterogeneity  = 0.09, I^2^ = 43.8%).

In one trial with 72 weeks and three trials with 96 weeks of follow-up the risk ratio in the intention to treat analysis of PI/r monotherapy compared to cART for viral suppression as defined in individual trials was 0.94 ((95%CI 0.87 to 1.03) p = 0.18, p for heterogeneity  = 0.87 I^2^ = 0%; risk difference −0.5 (95%CI −0.11 to 0.02) p = 0.18, p for heterogeneity 0.85, I^2^ = 0%). In three trials the risk ratios of viral suppression with <50 copies/ml of Pi/r monotherapy compared to cART were 0.95 ((95%CI 0.86 to 1.04) p>0.20, p for heterogeneity 0.71, I^2^  = 0%; risk differences −0.04 (95%CI −0.12 to 0.03) p>0.20, p for heterogeneity  = 0.70, I^2^ = 0%), and for viral suppression with <500 copies/ml 0.96 ((95%CI 0.85 to 1.07) p>0.20, p for heterogeneity  = 0.73, I^2^ = 0%; risk difference −0.04, (95%CI −0.13 to 0.05) p>0.20, p for heterogeneity  = 0.71, I^2^ = 0%).

In the per-protocol analysis of three trials with 72 to 96 weeks of follow-up the risk ratio of viral suppression as defined in individual trials of PI/r monotherapy compared to cART was 0.98 ((95%CI 0.93 to 1.03) p>0.20, p for heterogeneity  = 0.52 I^2^ = 0%; risk difference −0.02 (95%CI −0.07 to 0.03) p>0.20, p for heterogeneity 0.46, I^2^ = 0%). The risk ratio of PI/r monotherapy compared to cART for viral suppression with <50 copies/ml were 0.92 ((95%CI 0.85 to 0.99) p = 0.03. p for heterogeneity 0.63, I^2^ = 0%; risk difference −0.07 (95%CI −0.14 to 0.01) p = 0.02, p for heterogeneity  = 0.50, I^2^ = 0%) and for viral suppression with <500 copies/ml 0.98 ((95%CI 0.92 to 1.05) p>0.20, p for heterogeneity 0.36, I^2^ = 2.6%; risk difference −0.02 (95%CI −0.9 to 0.04) p>0.20, p for heterogeneity  = 0.29, I^2^ = 18.9%).

In sensitivity analysis we found for all virological endpoints similar effects sizes in subgroups of trials according to the type of PI (lopinavir versus darunavir) and indicators of trial quality (reporting of concealed patient allocation and power calculation for non-inferiority) with no significant interaction in any of the comparison pairs (data not shown).

### Other results

Of 44 patients with virological failure in monotherapy groups with reintroduction of NRTIs, 41 (93%) achieved again viral suppression. Four trials reported PI mutations in failing patients: There were seven patients with genotypic PI mutations with PI/r monotherapy and three patients with cART. In two patients with PI/r monotherapy the PI mutations had been detected already at baseline. Following switch to another PI/r or re-introduction of NRTIs all failing PI/r monotherapy patients with identified PI mutations were re-suppressed. Available data precluded the formal pooling of data on CD4 cell differences. None of the ten trials reported a statistically significant difference in change in CD4 cells between treatment groups. There were one death (not AIDS related) and one AIDS case in patients with PI/r monotherapy from one trial [40], [49]. There were no differences in severe adverse events in seven of nine trials reporting such data, but two trials reported a higher rate of discontinuation due to adverse events in cART arms [42], [48], [49].

## Discussion

This meta-analysis of ten randomized controlled trials indicates that PI/r monotherapy compared to cART with a PI/r and two NRTIs is associated with lower virological suppression and an increased risk of virological failure. This finding was consistent when we used three different definitions for virological failure. According to the virological endpoint used the absolute increase in risk of virological failure at 48 weeks with PI/r monotherapy compared to cART was in the intention to treat analyses between 2% and 6% and 13% at worst and in the per protocol analysis between 5% and 6% and 10% at worst. For trials with longer follow-up these estimates tended to be slightly worse. However, reintroduction of NRTIs lead in 93% of patients to de-novo viral suppression.

Our study has several strengths. It is based on a comprehensive search and the collaborative effort of the investigators of the primary studies to collect and present data of all included trials. We used well defined inclusion criteria and limited our analysis to patients on ritonavir boosted PI who were virologically well suppressed at baseline. Primary data provided by investigators allowed for sensitivity analysis with the use of different definitions for virological failure and we provide relative and absolute summary effects based on an intention to treat and per-protocol approach.

This study presents several limitations. The methodological quality of included trials was fair, although five trials did not report sample size statistics to test for non-inferiority of PI/r monotherapy, six trials did not report on concealment of treatment allocation, and all trials used an open design with unblinded outcome assessment. We made an effort to include all eligible trials but publication bias cannot be ruled out and testing for publication by use of funnel plot was uninformative due to the limited number of trials. We identified in registries one larger trial that was never published which is of concern. We were unable to evaluate other clinical endpoints we had specified in the protocol as change in CD4 cell count, elevations in lipid, hepatic and renal parameters. The sample size of the majority of included trials was small and additional trials will contribute to increase the precision of our summary estimates. We identified several ongoing trials that will roughly add 1300 patients to the analysis but cannot be expected before 2014. Given the recent interest in PI/r monotherapy, nevertheless, we believe that our findings are informative at this time.

We found heterogeneity for relative and absolute summary estimates but the limited number of trials did not allow to further explore differences in sensitivity analysis. Due to inconsistent reporting we were unable to formally pool CD4 cell measurements and safety data. None of the studies was powered for clinical events and the number of AIDS defining events or death was low.

In a comparative trial designed to show superiority of an experimental treatment the intention to treat analysis is conservative. When non-inferiority has to be shown, this is not the case, because any blurring of the difference between the treatment groups will increase the chances to declare equivalence [51]. Therefore, we conducted a per protocol analysis because the removal of uninformative patient data will increase our chances to detect any difference between the comparison groups. Our estimates in the intention to treat and per-protocol analyses were very similar, although upper bounds of 95% confidence intervals in the per-protocol analyses of all endpoints were further away from the point estimate of no effect.

We did not define a-priori a non-inferiority margin because subjectivity and judgement are involved in this determination. When choosing a non-inferiority margin a conservative approach that combines statistical reasoning and clinical judgement reflecting uncertainties in the evidence should be taken [52]. The fixed margin method fulfils these requirements where first a margin M_1_ to reflect the entire effect of the active comparator or control regimen is calculated from past trials. A margin M_2_ is then defined, the largest clinically accepted difference (degree of inferiority) of the test drug compared to the active control [53]. The active control effect in non-inferiority trials is not measured (there is no placebo), and therefore this effect must be assumed. When pooling treatment effects from six randomised trials comparing lopinavir based cART against cART regimens not containing lopinavir - the best available evidence to suit our needs - we may derive a relative risk margin M_1_ for virological suppression (<50 copies/ml) of 0.77 (data not shown). If we were to conclude that PI/r monotherapy would be necessary to preserve 50% of the conventional cART effect, the M_2_ relative risk margin would be 0.89, corresponding to a delta of 11% for a loss of effect to be ruled out. This estimate is conservative, because empirical evidence indicates that investigators tend to choose for surrogate marker endpoint trials higher non-inferiority margins [13], [14]. Most antiretroviral drug trials have defined a delta of 10% to 12% to reflect the largest difference in outcomes between treatment arms that could reasonably be assumed to be clinically equivalent [54].

Formally, the estimated risk differences for the <50 copies/ml cut-off indicated in both the intention to treat and per protocol analysis a higher risk difference for failure in patients with PI/r monotherapy that was of borderline significance whereas the corresponding relative summary estimates were not all statistically significant. The likely reasons for these discrepancies are variations in the underlying event rates in the control groups across trials. This meta-analysis and the included trial were formally designed to investigate non-inferiority and not superiority and therefore estimates for upper-bound confidence intervals should be disregarded.

In trials with 96 weeks of follow-up viral suppression rates by any definition tended to be lower in patients with PI/r monotherapy. Several trials reported a higher rate of intermittent viremia in patients with PI/r monotherapy [40], [45]. No clinically relevant differences in PI mutations were found in failing monotherapy patients when compared to patients failing with cART and re-intensification with two NRTIs was effective to regaining virological suppression in the overwhelming majority of patients. These findings are reassuring that PI/r monotherapy is a promising approach that should be further evaluated for long-term safety.

PIs have a poor central nervous system (CNS) penetration and data from one trial [41] indicated that patients with PI/r monotherapy have a higher rate of HIV replication in the liquor, and in some patients HIV replication in CNS was found even when HIV in serum was below 400 copies/ml, but this is a very rare finding. HIV replication in the CNS with clinical CNS symptoms in the presence of suppressed HIV in serum has been observed by others as well [55]. Five patients with PI/r monotherapy and confirmed HIV replication in the CNS from two trials were found to have CNS symptoms [41], [45]. In three of these patients symptoms were quite unspecific and may have been attributed to monotherapy in the context of the open trial design. PI/r monotherapy may reduce long-term side effects from NRTIs such as the risk of lipodystrophy [56], [57]. It is unclear whether PI/r monotherapy reduces the risk of bone mineral density loss and current evidence from clinical trials whether a NRTI sparing regimen conserves bone mineral density is conflicting [58], [59]. However, according to the PI used the risk of lipid anomalies is higher in comparison to a NNRTI based therapy. Whether PI/r monotherapy does reduce long-term side effects is subject of investigations of ongoing trials.

PI/r monotherapy may be associated with considerable cost savings. In this meta-analysis we did not include a model for an economic evaluation of the consequences of PI/r monotherapy and costs. In an economic analysis and simulation model Schackman et al. estimated the cost-effectiveness of a PI simplification strategy with boosted atazanavir compared to full cART based on efavirenz, tenofovir and emtricitabine [60]. In their analysis the average discounted lifetime costs for the simplification strategy was estimated to be US$ 430,200 for those without acquired PI resistance, $383,300 for those developing PI resistance and $ 456,700 for those on standard ART. The quality adjusted discounted life expectancy (QALE) for patients without PI resistance was higher (14.9 years) compared to standard care (14.7 years), however, patients with PI monotherapy and acquired PI resistance had an estimated QALE of 14.5 years. The assumptions for virological failure and risk of PI resistance that formed the base in their model are in comparison to findings from this meta-analysis overly pessimistic. Thus, the postulated gains in quality of life and cost savings of PI monotherapy seem conservative and would deserve confirmation in models with updated clinical data. Whether PI monotherapy is a cost-effective strategy in resource limited settings is unknown. Further economic studies evaluating in particular lopinavir in resource limiting settings are needed [61].

Our findings are informative for clinicians who wish to individualize antiretroviral therapy for HIV-infected patients with different preferences. Virologically well suppressed patients with excellent adherence may opt for PI/r monotherapy if they put a high value on avoiding long-term complications from their current NRTI backbone. The absolute increase in risk of virological failure at one year with PI/r monotherapy is roughly 10% to 13% at worst, with a very high chance of virological control when NRTIs are reintroduced in case of failure. PI monotherapy is not an option for clinicians and patients who do not want to accept this risk. Clinicians may also be hesitant and await the results from long-term follow-up data and more safety data in regard to HIV replication in the CNS before offering PI/r monotherapy for maintenance to their patients. When costs savings for antiretroviral therapy are a driving decision issue then PI/r monotherapy can be considered.

## Supporting Information

Figure S1 **Funnel plots of randomised controlled trials of proteinase inhibitor monotherapy versus combination antiretroviral therapy.** Intention to treat analysis; Virological failure as defined in individual trials, Egger's test for small study effect: p>.20.
(TIF)

Figure S2 **Funnel plots of randomised controlled trials of proteinase inhibitor monotherapy versus combination antiretroviral therapy.** Intention to treat analysis; Virological failure <50 copies/ml, Egger's test for small study effect: p>.10.
(TIF)

Figure S3 **Funnel plots of randomised controlled trials of proteinase inhibitor monotherapy versus combination antiretroviral therapy.** Intention to treat analysis; Virological failure <500 copies/ml, Egger's test for small study effect, p>.20.
(TIF)

Figure S4 **Funnel plots of randomised controlled trials of proteinase inhibitor monotherapy versus combination antiretroviral therapy.** Per protocol analysis; Virological failure as defined in individual trials, Egger's test for small study effect: p = .02.
(TIF)

Figure S5 **Funnel plots of randomised controlled trials of proteinase inhibitor monotherapy versus combination antiretroviral therapy.** Per protocol analysis; Virological failure <50 copies/ml, Egger's test for small study effect: p>.05.
(TIF)

Figure S6 **Funnel plots of randomised controlled trials of proteinase inhibitor monotherapy versus combination antiretroviral therapy.** Per protocol analysis; Virological failure <500 copies/ml, Egger's test for small study effect, p = .03.
(TIF)

Table S1 **Baseline characteristics of protease inhibitor monotherapy versus continued combination antiretroviral.**
(DOC)

Table S2 **Virological failure data in trials of protease inhibitor monotherapy versus continued combination antiretroviral therapy.**
(DOC)

Table S3 **Risk differences for virological failure of protease inhibitor monotherapy versus continued combination antiretroviral.** Therapy at 48 weeks of follow-up according to different virological endpoint definitions.
(DOC)
