# Supplementary figures and images for: Effectiveness of Protease Inhibitor Monotherapy versus Combination Antiretroviral Maintenance Therapy: A Meta-Analysis
Source: PLoS One. 2011 Jul 19;6(7):e22003. doi: 10.1371/journal.pone.0022003 (PMC3139616; doi:10.1371/journal.pone.0022003)

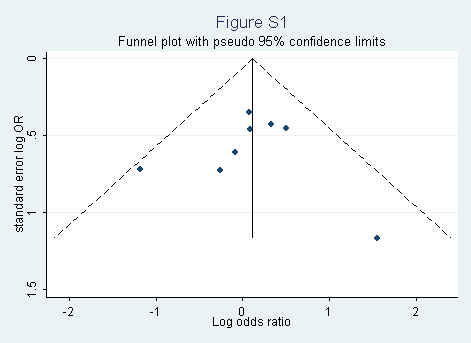

Supplement: Figure S1 — Funnel plots of randomised controlled trials of proteinase inhibitor monotherapy versus combination antiretroviral therapy. Intention to treat analysis; Virological failure as defined in individual trials, Egger's test for small study effect: p>.20. (TIF) [file pone.0022003.s001.tif]

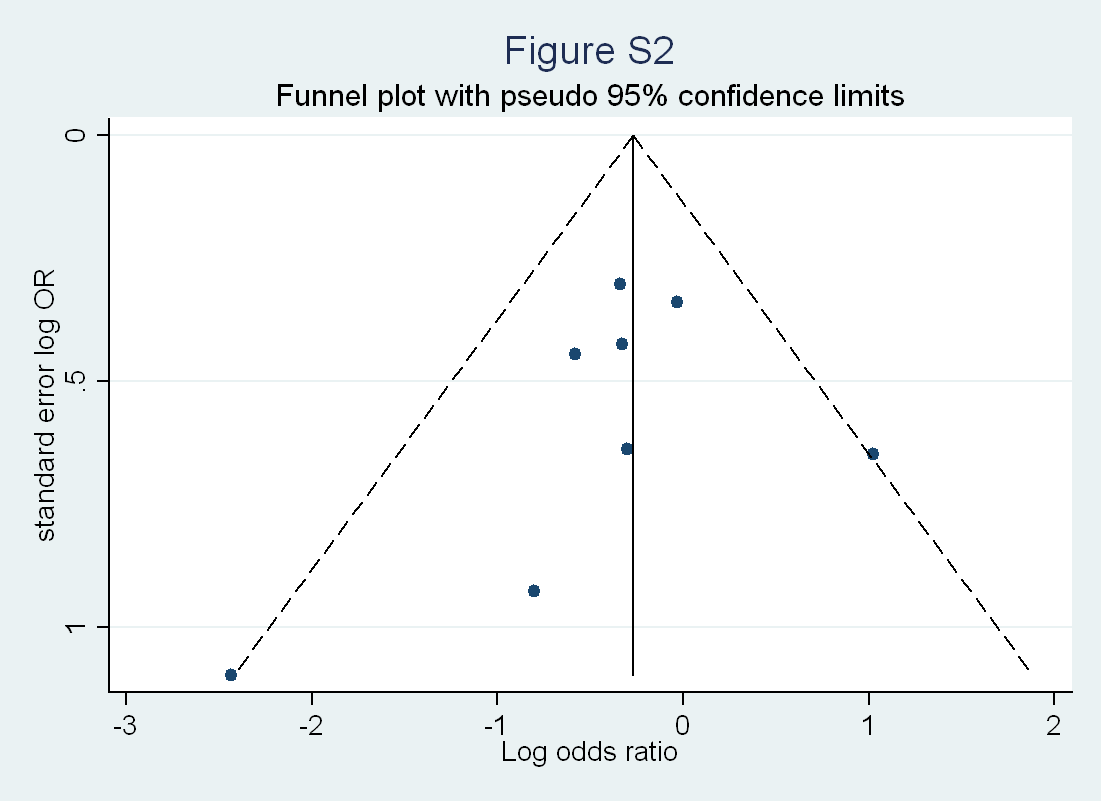

Supplement: Figure S2 — Funnel plots of randomised controlled trials of proteinase inhibitor monotherapy versus combination antiretroviral therapy. Intention to treat analysis; Virological failure <50 copies/ml, Egger's test for small study effect: p>.10. (TIF) [file pone.0022003.s002.tif]

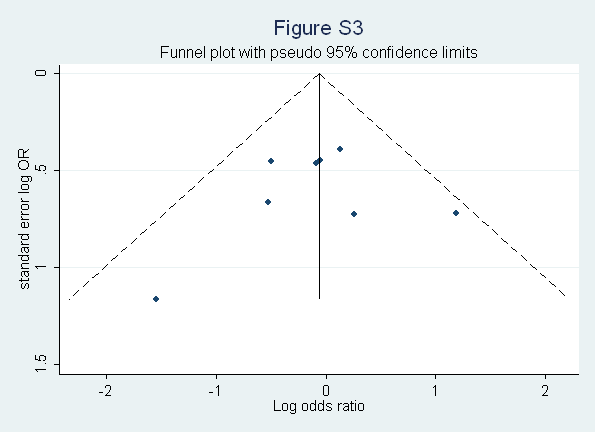

Supplement: Figure S3 — Funnel plots of randomised controlled trials of proteinase inhibitor monotherapy versus combination antiretroviral therapy. Intention to treat analysis; Virological failure <500 copies/ml, Egger's test for small study effect, p>.20. (TIF) [file pone.0022003.s003.tif]

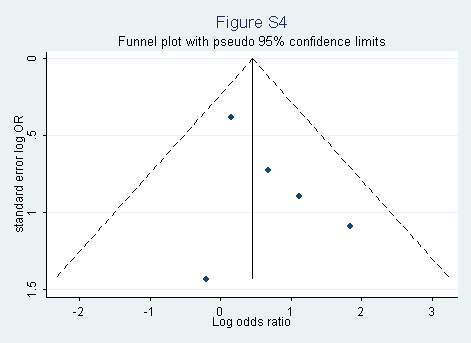

Supplement: Figure S4 — Funnel plots of randomised controlled trials of proteinase inhibitor monotherapy versus combination antiretroviral therapy. Per protocol analysis; Virological failure as defined in individual trials, Egger's test for small study effect: p = .02. (TIF) [file pone.0022003.s004.tif]

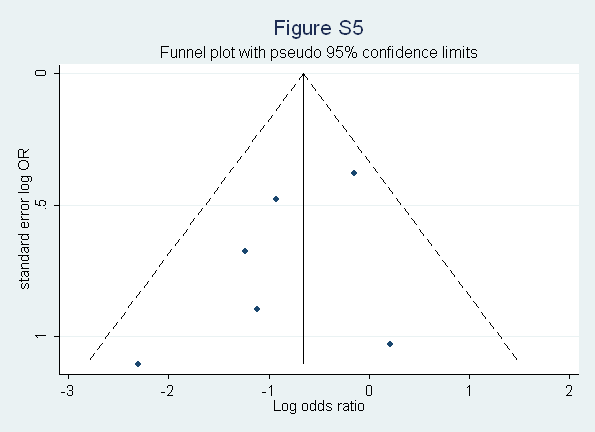

Supplement: Figure S5 — Funnel plots of randomised controlled trials of proteinase inhibitor monotherapy versus combination antiretroviral therapy. Per protocol analysis; Virological failure <50 copies/ml, Egger's test for small study effect: p>.05. (TIF) [file pone.0022003.s005.tif]

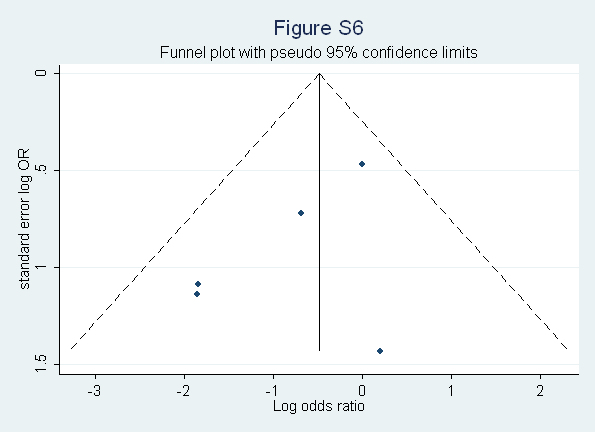

Supplement: Figure S6 — Funnel plots of randomised controlled trials of proteinase inhibitor monotherapy versus combination antiretroviral therapy. Per protocol analysis; Virological failure <500 copies/ml, Egger's test for small study effect, p = .03. (TIF) [file pone.0022003.s006.tif]
