# Supplementary material for: Effectiveness of Protease Inhibitor Monotherapy versus Combination Antiretroviral Maintenance Therapy: A Meta-Analysis
Source: PLoS One. 2011 Jul 19;6(7):e22003. doi: 10.1371/journal.pone.0022003 (PMC3139616; doi:10.1371/journal.pone.0022003)
Supplement: Table S1 — Baseline characteristics of protease inhibitor monotherapy versus continued combination antiretroviral. (DOC) [file pone.0022003.s007.doc]

Table S1 Baseline characteristics of protease inhibitor monotherapy versus continued combination antiretroviral therapy

| Baseline characteristics of trials | | | | | | | | | | Trial Validity | | | |
| --- | --- | --- | --- | --- | --- | --- | --- | --- | --- | --- | --- | --- | --- |
| Trial  Year of publication | Protease inihibitor | Subjects  (n) | Follow up (weeks) | Inclusion criteria | Age (years, mean*) | Males  n (%) | IV drug use  n (%) | Log viral load (copies/ml, mean*) | CD4 cells (per µL, mean*) | Allocation  concealed | Description of all loss to follow-up | loss to follow up ≤10% | Adequate power calculation for non inferiority |
| Arribas 2005 [4,48] | Lopinavir/r | I: 21  C: 21 | 72 | HAART with r/lopinavir for ≥4weeks, VL≤50 cps/ml for ≥6 months | I : 40  C: 40 | I:17(81)  C:18(86) | I:8(38)  C:6(29) | I:5.07  C: 5.01 | I:627  C:531 | yes | yes | yes | no |
| Pulido 2008 [40,49] | Lopinavir/r | I:100  C: 98 | 96 | HAART with r/lopinavir for ≥4weeks, VL<50 cps/ml for ≥6 months | I: 40  C: 42 | I:79(78)  C:84(82) | I:44(43)  C:47(46) | I:4.95  C:5.02 | I:521  CI490 | yes | yes | yes | yes |
| Echeverria 2007[47] | Saquinavir/r | I: 17  C: 11 | 48 | on HAART, VL<50 copies/ml for ≥6 months | I: 44  C: 42 | I:15(88)  C:11(100) | I:NR  C:NR | I:NR  C:NR | I:838  C:522 | NR | yes | yes | no |
| Cahn 2009 [43] | Lopinavir/r | I: 41  C: 39 | 52 | VL<50 copies/ml for ≥6 months, CD4 cells ≥100/mm³, on first HAART treatment regimen | I: 40  C: 38 | I:31(76)  C:36(92) | I:NR  C:NR | I:NR  C:NR | I:NR  C:NR | NR | yes | yes | no |
| Meynard 2010 [44] | Lopinavir/r | I:87   C:99 | 48 | VL<50 copies/ml for ≥6 months, no previous virological failure with a PI | I:43*  C:44* | I:63(72)  C:75(76) | I:NR  C:NR | I:NR  C:NR | I:NR  C:NR | yes | yes | yes | yes |
| Nunes 2007 [42] | Lopinavir/r | I: 30  C: 30 | 96 | VL<80 copies/ml, stable HAART for ≥6 months, CD4 cells >200/mm³, CD4 cells nadir >100/mm³ | I:40* C:39* | I:17(55)  C:20(69) | I:NR  C:NR | I:NR  C:NR | I:480*  C:528* | NR | yes | yes | no |
| Gutmann 2010 [41] | Lopinavir/r | I: 29  C: 31 | 48 | HAART >6 months, VL<50 copies/ml for ≥3 months | I:42  C:46 | I:19(66)  C:24(77) | I:NR  C:NR | I:49  C:48 | I:160  C:160 | NR | yes | yes | yes |
| Waters 2008 [39] | Lopinavir/r | I: 26  C: 28 | 48 | HAART for >6 months, CD4 cells ≥200/mm³, VL <400 copies/ml for 18 months and VL<50 copies/ml at study entry | I:43  C:46 | I:23(88)  C:24(86) | I:NR  C:NR | I:1.79  C:1.7 | I:576  C:486 | NR | no | yes | no |
| Arribas 2010 [46,50] | Darunavir/r | I:127  C:129 | 96 | HAART at screening, VL<50 copies/ml for ≥6 months, no prior use of darunavir | I:43  C:44 | I:99(78)  C:107(83) | I:20(16)  C:11(9) | I:NR  C:NR | I:571*  C:579* | yes | yes | yes | yes |
| Katlama 2009 [45] | Darunavir/r | I: 112  C: 113 | 48 | cART for ≥18 months, and VL<400 copies, VL<50 copies/ml at entry, CD4 count ≥200cells/m³ | I:46*  C:45* | I:83(74)  C:87(77) | I:10 (9)  C:3(3) | I:4.9*  C:4.9* | I:585*  C:582* | NR | yes | yes | yes |
| I Intervention group (Protease inhibitor mono therapy), C Control Group (Continued 2 class antiretroviral therapy), NA Not applicable, NR Not reported* medians if means were not reported, VL viral load | | | | | | | | | | | | | |
