# Supplementary material for: Effectiveness of Protease Inhibitor Monotherapy versus Combination Antiretroviral Maintenance Therapy: A Meta-Analysis
Source: PLoS One. 2011 Jul 19;6(7):e22003. doi: 10.1371/journal.pone.0022003 (PMC3139616; doi:10.1371/journal.pone.0022003)
Supplement: Table S2 — Virological failure data in trials of protease inhibitor monotherapy versus continued combination antiretroviral therapy. (DOC) [file pone.0022003.s008.doc]

Table S2 Virological failure data in trials of protease inhibitor monotherapy versus continued combination antiretroviral therapy

| Trial  Year of publication | Definition of virological failure | HIV Assay | Number of subjects | Loss of virological suppression* (n) | Discontinuation of study drug or loss to follow-up (n) | Failure by ITT^±^ (n) | Viral load <50 copies/ ml at week 48 | Viral load <500 copies/ ml at week 48 | Number of re-intensifications after failure* (and patients with second failure^±^) |
| --- | --- | --- | --- | --- | --- | --- | --- | --- | --- |
| Arribas 2005 [4,48] | 2 consecutive measurements of plasma HIV-1 RNA>500 copies/ml | Ampliprep/Cobas Amplicor Monitor (Roche) | I: 21  C: 21 | I:3  C:0 | I:1  C:1 | I:4  C:1 | I:17  C:20 | I:17  C:20 | 3(0) |
| Pulido 2008 [40] | 2 consecutive measurements of plasma HIV-1 RNA>500 copies/ml | Cobas Ampliprep/Cobas Taqman (Roche) | I:100  C: 98 | I:6  C:3 | I:5  C:7 | I:11  C:10 | I:85  C:88 | I:89  C:88 | 12(2) |
| Echeverria 2007 [47] | 2 consecutive measurements of plasma HIV-1 RNA>50 copies/ml | Information missing | I: 17  C: 11 | I:1  C:0 | I:3  C:0 | I:4  C:0 | I:13  C:11 | I:NR  C:NR | 1(0) |
| Cahn 2009 [43] | 2 consecutive measurements of plasma HIV-1 RNA>200 copies/ml | Information missing | I: 41  C: 39 | I:1  C:0 | I:2  C:8 | I:3  C:8 | I:37  C:30 | I:38^#^  C:31^#^ | 4(0) |
| Meynard 2010 [44] | 2 consecutive measurements of plasma HIV-1 RNA>50 copies/ml | Information missing | I:87   C:99 | I:5  C:0 | I:0  C:7 | I:14  C:12 | I:73  C:87 | I:76^$^  C:87^$^ | 6(0) |
| Nunes 2007 [42] | 2 consecutive measurements of plasma HIV-1 RNA>500 copies/ml | NASBA assay | I: 30  C: 30 | I:1  C:1 | I:3  C:4 | I:4  C:5 | I:26^¶^  C:25^¶^ | I:26^†^  C:25^†^ | 1(0) |
| Gutmann 2010 [41] | 2 consecutive measurements of plasma HIV-1 RNA>400 copies/ml | Cobas Ampiprep/Cobas Taqman (Roche) | I: 29  C: 31 | I:5  C:0 | I:1  C:0 | I:6  C:0 | I:NR  C:NR | I:23^†^  C:31^†^ | 6(0) |
| Waters 2008 [39] | 2 consecutive measurements of plasma HIV-1 RNA>50 copies/ml | Information missing | I: 26  C: 28 | I:6  C:4 | I:2  C:4 | I:7  C:8 | I:18  C:22 | I:18^#^  C:23^#^ | 0(0) |
| Arribas 2010 [46] | 2 consecutive measurements of plasma HIV-1 RNA>50 copies/ml | Roche Amplicor | I:127  C:129 | I:11  C:7 | I:9  C:11 | I:20  C:19 | I:107  C:110 | I:113  C:113 | 7 (0) ‡ |
| Katlama 2009 [45] | 2 consecutive measurements of plasma HIV-1 RNA>400 copies/ml | Information missing | I: 112  C: 113 | I:3  C:0 | I:3  C:3 | I:14  C:9 | I:97  C:104 | I:98^$^  C:104^$^ | 3(0) |
| * As defined in individual trials ± Any switch or loss to follow = failure ±Second failure is defined with viral load >50 copies/ml ‡ by 48 weeks  ^¶^ viral load <80 copies/ml, ^†^ viral load <1000 copies/ml, ^#^ viral load <200 copies/ml, ^$^ viral load <400 copies/ml  HIV Assay | | | | | | | | | |
