# Supplementary material for: Effectiveness of Protease Inhibitor Monotherapy versus Combination Antiretroviral Maintenance Therapy: A Meta-Analysis
Source: PLoS One. 2011 Jul 19;6(7):e22003. doi: 10.1371/journal.pone.0022003 (PMC3139616; doi:10.1371/journal.pone.0022003)
Supplement: Table S3 — Risk differences for virological failure of protease inhibitor monotherapy versus continued combination antiretroviral. Therapy at 48 weeks of follow-up according to different virological endpoint definitions. (DOC) [file pone.0022003.s009.doc]

Table S3 Risk differences for virological failure of protease inhibitor monotherapy versus continued combination antiretroviral

Therapy at 48 weeks of follow-up according to different virological endpoint definitions

| Trial  Year of publication | Intention to treat (loss of follow, switch equals failure) | | | | Per-protocol analysis | | |
| --- | --- | --- | --- | --- | --- | --- | --- |
|  | Virological failure as defined in trial  (95%CI) | Viral load <50 copies ml/  (95%CI) | Viral load <500 copies / ml  (95%CI) | Proportion with loss of viral suppression as defined in trial  (95%CI) | Virological failure as defined in trial  (95%CI) | Viral load <50 copies ml/  (95%CI) | Viral load < 500 copies / ml  (95%CI) |
| Arribas 2005 [4,48] | -0.14  (-0.33 to0.05) | -0.14  (-0.33 to 0.05) | -0.14  (-0.33 to 0.05) | -0.14  (-0.31 to 0.02) | -0.15  (-0.32 to 0.02) | -0.15  (-0.32 to 0.02) | -0.15  (-0.32 to 0.02) |
| Pulido 2008 [40] | -0.01  (-0.09 to 0.08) | -0.05  (-0.14 to 0.04) | -0.01  (-0.09 to 0.08) | -0.03  (-0-09 to 0.03) | -0.03  (-0.09 to 0.03) | -0.07  (-0.14 to 0 ) | -0.03  (-0.09 to 0.03) |
| Echeverria 2007 [47] | -0.24  (-0.47 to -0.01) | -0.24  (-0.47 to -0.01) |  | -0.06  (-0.23 to 0.11) | -0.07  (-0.26 to 0.12) | -0.07  (-0.26 to 0.12) |  |
| Cahn 2009 [43] | 0.13  (-0.02 to 0.28) | 0.13  (-0.03 to 0.29) | 0.13  (-0.02 to 0.28) ^#^ | -0.02  (-0.09 to 0.04) | 0.01  (-0.07 to 0.08) | 0.01  (-0.10 to 0.12) | 0.01  (-0.07 to 0.08) ^#^ |
| Meynard 2010 [44] | 0.04  (-0.06 to 0.14) | 0.04  (-0.06 to 0.14) | 0.01  (-0.09 to 0.10) ^$^ | 0.06  (0.01 to 0.11) | 0.07  (0.01 to 0.12) | 0.07  (0.01 to 0.12) | 0.01  (-0.02 to 0.05) ^$^ |
| Nunes 2007 [42] | 0.03  (-0.15 to 0.21) |  | 0.03  (-0.15 to 0.21) ^†^ | 0.00  (-0.09 to 0.09) |  |  |  |
| Gutmann 2010[41] | -0.21  (-0.36 to 0.05) | -0.24  (-0.42 to -0.07) | -0.21  (-0.36 to -0.05) | -0.17  (-0.32 to -0.03) | -0.18  (-0.33 to -0.03) | -0.22  (-0.39 to -0.05) | -0.18  (-0.33 to -0.03) |
| Waters 2008 [39] | 0.02  (-0.22 to 0.26) | -0.06  (-0.28 to 0.17) | -0.09  (-0.31 to 0.13) ^#^ | -0.09  (-0.30 to 0.12) | -0.13  (-0.34 to 0.07) | -0.13  (-0.34 to 0.07) | -0.18  (-0.35 to 0.01) ^#^ |
| Arribas 2010 [46] | -0.01  (-0.10 to 0.08) | -0.01  (-0.10 to 0.08) | 0.01  (-0.07 to 0.09) | -0.03  (-0.10 to 0.03) | -0.02  (-0.10 to 0.07) | -0.02  (-0.10 to 0.07) | 0.  (-0.07 to 0.07) |
| Katlama 2009 [45] | -0.05  (-0.12 to 0.03) | -0.05  (-0.14 to 0.03) | -0.05  (-0.12 to 0.03) ^$^ | -0.03  (-0.06 to 0.01) | -0.05  (-0.10 to 0) | -0.09  (-0.17 to 0) | 0.05  (0 to 0.10) ^$^ |
| Pooled estimates  [P for heterogenity, I^2^] | -0.04  (-0.09 to 0.02) p=0.16  (P=0.08, I^2^= 41.2%) | -0.06  (-0.11 to 0)  p=0.05  (P=0.08, I^2^= 43.1%) | -0.02  (-0.08 to 0.03)  P=0.37  (P=0.10, I^2^= 39.6%) | -0.04  (-0.06 to -0.02)  P=0.04  (P=0.55, I^2^= 0.0%) | -0.05  (-0.08 to -0.02)  P<0.01  (P=0.42, I^2^= 2.2%) | -0.07  (-0.10 to -0.03)  P<0.01  (P=0.44, I^2^= 0.0%) | -0.04  (-0.07 to 0)  P=0.04  (P=0.09, I^2^= 43.8%) |
| ^†^ viral load <1000 copies/ml, ^#^ viral load <200 copies/ml, ^$^ viral load <400 copies/ml | | | | | | | |
